# Preparation and Gas-Sensing Properties of Two-Dimensional Molybdenum Disulfide/One-Dimensional Copper Phthalocyanine Heterojunction

**DOI:** 10.3390/s23239321

**Published:** 2023-11-22

**Authors:** Guoqing Chen, Xiaojie Xu, Hao Wang, Talgar Shaymurat

**Affiliations:** Key Laboratory of New Energy and Materials Research, Xinjiang Institute of Engineering, Urumqi 830023, China; cgq08250624@163.com (G.C.); 18099415421@163.com (X.X.); wh18299190475@163.com (H.W.)

**Keywords:** MoS_2_, CuPc, heterojunction, gas sensor, VOC, ethanol

## Abstract

Although 2D MoS_2_ alone shows excellent gas-sensing performance, it is prone to stacking when used as the sensitive layer, resulting in insufficient contact with the target gas and lower sensitivity. To solve this, a 2D-MoS_2_/1D-CuPc heterojunction was prepared with different weight ratios of MoS_2_ nanosheets to CuPc micro-nanowires, and its room-temperature gas-sensing properties were studied. The response of the 2D-MoS_2_/1D-CuPc heterojunction to a target gas was related to the weight ratio of MoS_2_ to CuPc. When the weight ratio of MoS_2_ to CuPc was 20:7 (7-CM), the gas sensitivity of MoS_2_/CuPc composites was the best. Compared with the pure MoS_2_ sensor, the responses of 7-CM to 1000 ppm formaldehyde (CH_2_O), acetone (C_3_H_6_O), ethanol (C_2_H_6_O), and 98% RH increased by 122.7, 734.6, 1639.8, and 440.5, respectively. The response of the heterojunction toward C_2_H_6_O was twice that of C_3_H_6_O and 13 times that of CH_2_O. In addition, the response time of all sensors was less than 60 s, and the recovery time was less than 10 s. These results provide an experimental reference for the development of high-performance MoS_2_-based gas sensors.

## 1. Introduction

Due to industrial development, large amounts of toxic and harmful gases pollute the environment and endanger people’s health [1,2]. Thus, there is a need to control and monitor air pollution in real time. Common harmful gases include volatile organic compounds (VOCs), nitrogen oxides, sulfur oxides, and carbon oxides, which can damage a person’s sense of smell, vision, mucosa, lung function, liver function, and central nervous system [3,4]. These gases are produced mainly during industrial production, transportation, and household activities [5].

Numerous strategies have been developed for air-quality monitoring in recent years, including semiconductor gas sensors, which are detection devices with high sensitivity, a fast response speed, and low cost [6,7]. Commonly used sensing materials to construct semiconductor gas sensors include zero-dimensional [8], one-dimensional [9], two-dimensional [10], and three-dimensional [11] materials. Layered molybdenum disulfide (MoS_2_) is a two-dimensional (2D) material that has been widely studied because of its photoelectric properties, stable chemical properties, and simple preparation process [12,13,14]. Two-dimensional MoS_2_ has an ultra-high specific surface area, abundant surface defects, a tunable band gap (~1.8 eV), and excellent carrier mobility. These properties give it a high affinity for most harmful gas molecules [15,16], making it highly suitable for gas detection and gas sensor research. In the past decade, many studies have reported the gas-sensing properties of 2D MoS_2_ and its composites. Hai et al. obtained monolayer and multilayered MoS_2_ films via a mechanical peeling method using transparent tape. They prepared field-effect transistors (FETs) for NO gas detection for the first time by using photolithography. When 2–4 layers of MoS_2_ were used, the FET was stable and showed a LOD of 0.8 ppm for NO, but the stability when using a MoS_2_ monolayer was poor [17]. Li et al. prepared MoS_2_ via a hydrothermal method and obtained resistive devices. At an operating temperature of 100 °C, the device showed good selectivity for carbon monoxide (CO) and hydrogen sulfide (H_2_S), showing good repeatability and linearity [18]. Li Wenbo et al. prepared three different morphologies of molybdenum disulfide sensors for ammonia detection via a hydrothermal method. The results showed that the nanoflower-like MoS_2_ sensor had a better response to ammonia and showed excellent repeatability, stability, and selectivity, with a response of 7.41% and a recovery time of 874 s toward NH_3_ at 10 ppm. The response to NH_3_ was 4.5 times as high as methanol and isopropanol and 8 times higher than acetone. Under the same conditions, the response values of the other two morphologies were 2.01% and 5.11%, respectively [19].

The above studies showed that although 2D-MoS_2_ showed excellent gas-sensing performance when used alone, it was prone to stacking when used as the sensitive layer. This resulted in insufficient contact with the target gas, which reduced the sensitivity of the sensor and prolonged the response-recovery time of the sensor. Building on the research on 2D-MoS_2_ sensors, researchers have found that constructing heterojunctions is a feasible research method for solving the above shortcomings. The heterostructure formed of composite materials and MoS_2_ nanosheets can inhibit the interlayer agglomeration of MoS_2_ sheets, thereby providing sufficient active sites for the adsorption of gas molecules. This can help improve the gas detection performance of MoS_2_-based sensors [20]. Bu Xin et al. prepared MoS_2_/Cd_2_SnO_4_ composites via water heating–calcination and studied the effect of MoS_2_ doping on the gas-sensing properties. The experimental results showed that when the mass ratio between MoS_2_ and Cd_2_SnO_4_ was 2.5%, the response of the gas-sensing element prepared using MoS_2_/Cd_2_SnO_4_ composites to 100 ppm formaldehyde gas was 40 at 170 °C. This was 29 times that of pure MoS_2_ for the same concentration of formaldehyde gas [21]. Zhang et al. prepared CeO_2_/MoS_2_ composites via a hydrothermal method, which exhibited a better room-temperature NO_2_-sensing response and a 69.1% higher sensitivity to 5 ppm NO_2_ than pure MoS_2_. In total, 85% of the resistance was recovered within 900 s, and the sensor showed a fast response, long-term stability, and selectivity [22]. Hemalatha et al. designed an NH_3_ sensor consisting of two-dimensional transition-metal dichalcogenide (mainly WS_2_ and MoS_2_), and polyaniline (PANI). The test results showed that the WS_2_/PANI and MoS_2_/PANI composites had higher sensitivity to NH_3_ than individual MoS_2_ and PANI. The response and recovery times of the composite films were 10/16 s and 14/16 s, respectively [23]. 

The above results show that 2D MoS_2_ composites have better gas-sensing performance than monolayer MoS_2_ but still have the following problems when used in traditional composite thin-film sensors: (1) the thin-film material is highly ordered, which is not conducive to gas diffusion in the membrane and affects the response speed of the sensor; (2) they have a low flexibility, which affects the practical applications of the sensor. Most research has used inorganic nanomaterials and 2D-MoS_2_ composites, but there has been no research on organic one-dimensional (1D) materials and 2D-MoS_2_ composites. Copper phthalocyanine (CuPc) is a typical organic semiconductor material [24] with good thermal and chemical stability. Its structure can be easily modified, and it has been widely used to design and research thin-film gas sensors with good selectivity and low operating temperatures. Moreover, one-dimensional CuPc exhibits better gas-sensing properties than bulk CuPc. Therefore, a heterojunction strategy was used to improve the performance of gas sensors by combining the advantages of 2-D MoS_2_ and 1-D CuPc. 

Here, we report a gas sensor based on a heterojunction between synthetic CuPc micro-nanowires and exfoliated MoS_2_ nanosheets. One-dimensional CuPc was prepared via physical vapor deposition (PVD), and MoS_2_/CuPc composites with different mass ratios were prepared via a magnetic reflux stirring method. The performance of 2D-MoS_2_/1D-CuPc heterojunction gas sensing was studied using XRD, SEM, XPS, FTIR, and Raman spectroscopy.

## 2. Materials and Methods

### 2.1. Materials

Molybdenum disulfide (MoS_2_) and copper phthalocyanine (CuPc) were purchased from Sigma Aldrich. Potassium sulfate (K_2_SO_4_) was purchased from Xuzhuangzi, Dongli District, Tianjin. Absolute ethanol (C_2_H_6_O), acetonitrile (CH_3_CN), *N*-methyl pyrrolidone (C_5_H_9_NO), formaldehyde (CH_2_O), and acetone (C_3_H_6_O) were purchased from Tianjin Zhiyuan Chemical Reagent Co., Ltd. (Tianjin, China). The above chemicals were of analytical grade (AR) and did not require further purification.

### 2.2. Synthesis of MoS_2_ Nanosheets

MoS_2_ nanosheets were prepared via grinding-assisted liquid phase stripping [25]. First, 100 mg of MoS_2_ raw material was weighed and ground in an agate mortar for 2 h, and an appropriate amount of NMP was added during the grinding process. After that, the samples were placed in a vacuum oven and dried for 12 h after grinding. After drying, the samples were dispersed in 45 vol% absolute ethanol for 1 h, and then, the dispersion was centrifuged for 20 min (1500 r/min) to obtain MoS_2_ nanosheets. Finally, the sample was dried in air for later use (Figure S1a). 

### 2.3. Preparation of 1D-CuPc 

CuPc (copper phthalocyanine) micro-nanowires (1D-CuPc) were prepared via physical vapor deposition (PVD) [26], as shown in Figure S1b. Purified CuPc (1 mg) was placed in a beaker, 20 mL ethanol was added, and the beaker was sealed and sonicated for 1 h to form a CuPc suspension in ethanol. The suspension was left to stand for 24 h at room temperature to obtain a supernatant for later use. A silicon wafer substrate was clamped with forceps and immersed in the supernatant so that the nanoparticles in the suspension were transferred to the substrate. The nanoparticles acted as crystal nuclei on the substrate surface to induce the growth of 1D-CuPc. Materials with a length of more than 100 μm were obtained at a growth time of 4 h and a growth temperature of 400 °C, and using N_2_ as the carrier gas at a flow rate of 20 mL/min.

### 2.4. Preparation of 2D-MoS_2_/1D-CuPc Heterojunction 

Composites were prepared via heating with magnetic reflux stirring. The prepared MoS_2_ nanosheets (20 mg) were dispersed in 20 mL of absolute ethanol. Then, CuPc micro-nanowires were added, subjected to magnetic reflux stirring, and heated for 2 h. Finally, 2D-MoS_2_/1D-CuPc heterojunction samples were obtained via centrifugation at 8000 r/min. A series of 2D-MoS_2_/1D-CuPc heterojunction samples at different mass ratios were synthesized using the same method. The preparation of the 2D-MoS_2_/1D-CuPc heterojunction is shown in Figure S1. The weight ratios of MoS_2_ to CuPc were controlled at 20:3, 20:5, 20:7, 20:10, and 20:20, and the samples were named 3-CM, 5-CM, 7-CM, 10-CM, and 20-CM, respectively.

### 2.5. Material Characterization

A Raman spectrometer (inVia Renishaw) was used to analyze phases and the vibrational characteristics of the sample. The structures of all the samples were measured via X-ray diffraction (XRD) (Bruker D8 Advance, with Cu-K*α* radiation), and Fourier-transform infrared (FTIR) spectrometry (Bruker VERTEX 70, Saarbrücken, Germany). Field emission scanning electron microscopy (JSM-7610F Plus) was used to study the topography of the sample. The surface chemistry of the sample was measured via X-ray photoelectron spectroscopy (XPS K-Alpha+, Thermo Fisher Scientific, Waltham, MA, USA).

### 2.6. Gas-Sensing Performance Evaluation

First, 2D-MoS_2_/1D-CuPc heterojunctions with different mass ratios (20:3, 20:5, 20:7, 20:10, and 20:20) were dispersed in absolute ethanol at a concentration of 10 mg/mL. Then, the dispersions were used to carry out uniform coating (2 μL MoS_2_/CuPc composite dispersion) to fabricate a 2D-MoS_2_/1D-CuPc-based sensing chip (13 mm × 7 mm, 0.5 mm thick) with Ag-Pd interdigital electrodes (IDEs). The minimum width and spacing of the electrodes was 0.2 mm). Finally, the interdigital electrode was dried at 60 °C and aged for 48 h at a voltage of 2 V to obtain a sensing chip with good stability (Figure S1c).

**Figure 1:**
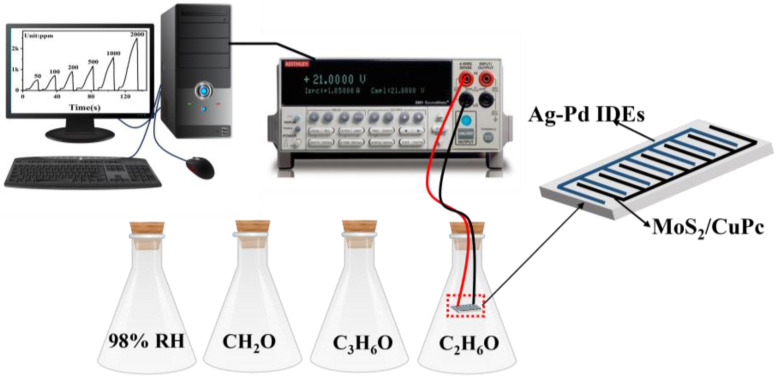
Schematic of sensing test system.

In this experiment, ethanol (C_2_H_6_O), acetone (C_3_H_6_O), and formaldehyde (CH_2_O) solutions were used to obtain 1000 ppm C_2_H_6_O, C_3_H_6_O, and CH_2_O gas environments, respectively. In addition, a 98%-relative-humidity environment was obtained using KNO_3_-saturated saline solution. The gas-sensing performance of the sensors was evaluated using a constant voltage of 2 V at 24 °C using a Keithley 2636b workstation (Keithley Instruments, Cleveland, OH, USA). The gas sensors were placed in different types of gas environments for testing, as shown in Figure 1. When the current stabilized, we switched to the next gas environment to continue the test. The current response of the gas performance test was defined as *Response* = (*I*_G_–*I*_R_)/*I*_R_, where *I*_R_ and *I*_G_ are the current of the sensor in the reference gas and the target gas, respectively. The response time was defined as the time period during which the sensor current reached 90% of the response value after exposure to the target gas, and the recovery time was defined as the time period when the sensor current become 10% of the response value after the target gas was removed.

## 3. Results and Discussion

### 3.1. Material Characterization and Analysis

**Figure 2:**
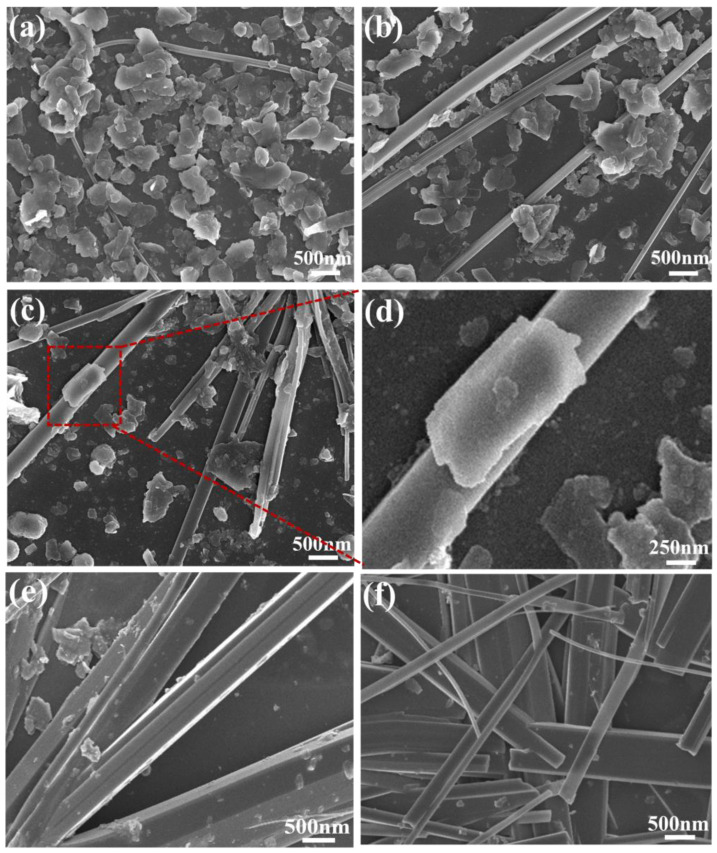
SEM image of 2D-MoS_2_/1D-CuPc heterojunction: (**a**) a weight ratio of 20:3 (3-CM); (**b**) a weight ratio of 20:5 (5-CM); (**c**,**d**) a weight ratio of 20:7 (7-CM); (**e**,**f**) weight ratios of 20:10 (10-CM) and 20:20 (20-CM), respectively.

The surface morphology of the 2D-MoS_2_/1D-CuPc heterojunction was characterized via SEM. As shown in Figure 2a–f, the morphologies of all samples were composed of MoS_2_ nanosheets and CuPc. The different mass ratios of MoS_2_ and CuPc changed the morphology of the 2D-MoS_2_/1D-CuPc heterojunction samples. Figure 2a shows the morphology of 3-CM, in which MoS_2_ nanosheets wrapped CuPc micro-nanowires, and MoS_2_ nanosheets were the main material. Figure 2b shows the morphology of 5-CM. Upon increasing the higher content of 3-CM CuPc micro-nanowires, MoS_2_ nanosheets were stacked on CuPc micro-nanowires. Figure 2c shows the morphology of 7-CM, in which MoS_2_ nanosheets penetrated the network of CuPC micro-nanowires, which should have allowed gas molecules to fully contact the material, and thus, improved the gas-sensing performance. Figure 2d shows that the MoS_2_ nanosheets were tightly bonded to the CuPC micro-nanowires to form a 2D-MoS_2_/1D-CuPc heterojunction. Figure 2e,f show 10-CM and 20-CM, respectively, which both had roughly the same structure, in which CuPc micro-nanowires were stacked to form conductive micro-nanowires.

**Figure 3:**
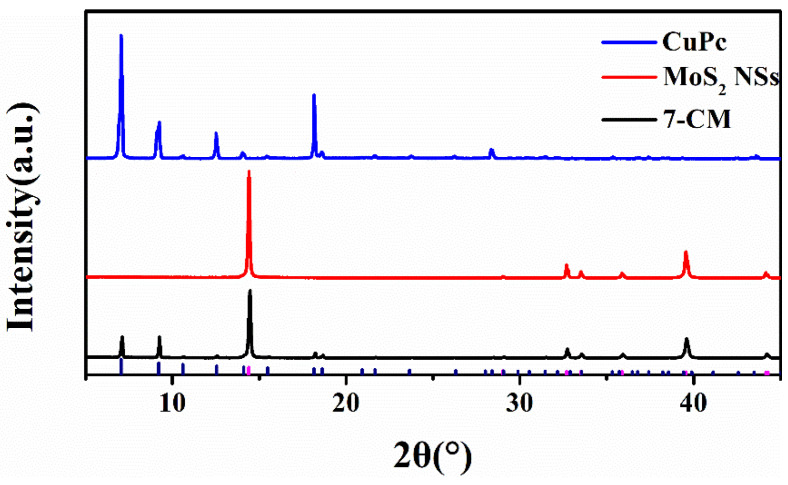
XRD patterns of CuPc, MoS_2_ NSs, and 2D-MoS_2_/1D-CuPc heterojunction (7-CM).

**Figure 4:**
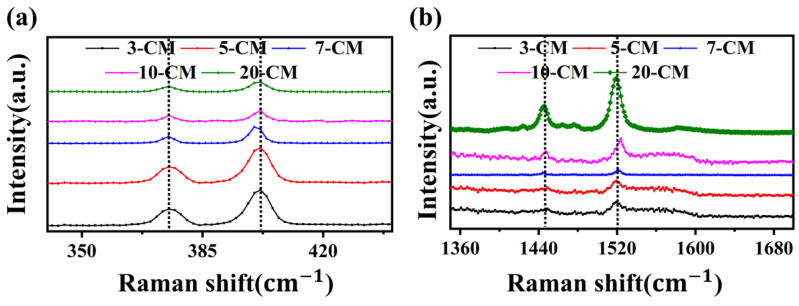
Raman spectra of 3-CM, 5-CM, 7-CM, 10-CM, and 20-CM. (**a**) Enlargement of the MoS_2_ peaks $E_{2g}^{1}$ and $A_{1g}$ and (**b**) Enlargement of the MoS_2_ peaks from 1360 cm^−1^ to 1680 cm^−1^.

Figure 3 shows the X-ray diffraction (XRD) patterns of CuPc micro-nanowires, MoS_2_ nanosheets, and 2D-MoS_2_/1D-CuPc (7-CM), which were used to determine the crystallinity and phase structure of the samples. Typical diffraction peaks at 7° and 9.2° were observed in the 2D-MoS_2_/1D-CuPc pattern, which corresponded to the (−1,0,1) and (1,0,1) crystal planes of β-phase CuPc, respectively. The diffraction peaks at 14.4°, 32.6°, 33.5°, 35.8°, 39.5°, 44.2°, and 49.8° corresponded to the (0,0,2), (1,0,0), (1,0,1), (1,0,2), (1,0,3), (1,0,4), and (1,0,5) crystal planes of MoS_2_. The results in Figure 4 show that CuPc was compounded onto the MoS_2_ surface [27].

Figure 4 shows the Raman spectra of 3-CM, 5-CM, 7-CM, 10-CM, and 20-CM, in which the strong signals at 375.3 cm^−1^ and 401.9 cm^−1^ in Figure 4a were attributed to the in-plane $E_{1}^{2g}$ and out-of-plane $\;A_{1g}$ vibration modes [28]. The characteristic peaks of the 7-CM out-of-plane$\;A_{1g}$ vibration mode underwent a blue shift and their intensity was reduced, indicating that the MoS_2_ nanosheets had fewer layers than the other samples. In Figure 4b, the strong signals at 1445.7 cm^−1^ and 1520.2 cm^−1^ were attributed to the asymmetric vibration of C=N and the telescopic vibration of C=C [29], either due to the presence of CuPc, residual NMP, or their combination.

**Figure 5:**
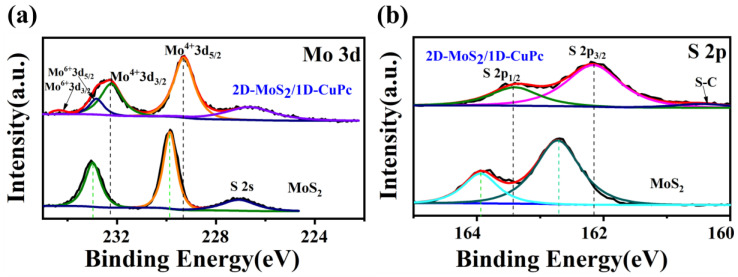
XPS spectra of (**a**) Mo 3d and (**b**) S 2p of the different samples.

The elemental composition and valence states of the composites were tested via XPS. It is obvious that MoS_2_ was composed of Mo, S, C, and O, and CuPc was composed of C, O, N, and Cu. The 2D-MoS_2_/1D-CuPc heterojunction was composed of Mo, S, Cu, C, and N elements (Figure S2). Figure 5a shows the Mo 3d spectra of the 2D-MoS_2_ nanosheet and the 2D-MoS_2_/1D-CuPc heterojunction. For the pure MoS_2_ nanosheet, two peaks can be seen at 232.21 eV and 229.27 eV, which correspond to Mo 3d_3/2_ and Mo 3d_5/2_, respectively [30]. In addition, the two faint peaks at 232.82 eV and 236.32 eV are typical signals of Mo^6+^ 3d_5/2_ and Mo^6+^ 3d_3/2_ in Mo-O bonds, respectively, suggesting that the introduction of O was caused by defects or vacancies on the surface of MoS_2_ [31]. The overall shape of the 2D-MoS_2_/1D-CuPc heterojunction’s Mo 3d spectrum was almost identical to that of pure MoS_2_ nanosheets. However, all peaks moved to lower binding energies, suggesting an increase in the electron cloud density around MoS_2_. A shift to lower binding energies was also found in the S 2p spectrum (Figure 5b). The results showed that the pure MoS_2_ nanosheets had two typical peaks at 162.15 eV and 163.40 eV, attributed to S 2p_3/2_ and S 2p_1/2_, respectively, while the 2D-MoS_2_/1D-CuPc heterojunction had peaks at lower binding energies of 162.73 eV and 163.96 eV [9]. Based on the above test results, it can be inferred that the superior interfacial contact at the 2D-MoS_2_/1D-CuPc heterojunction effectively transferred electrons.

**Figure 6:**
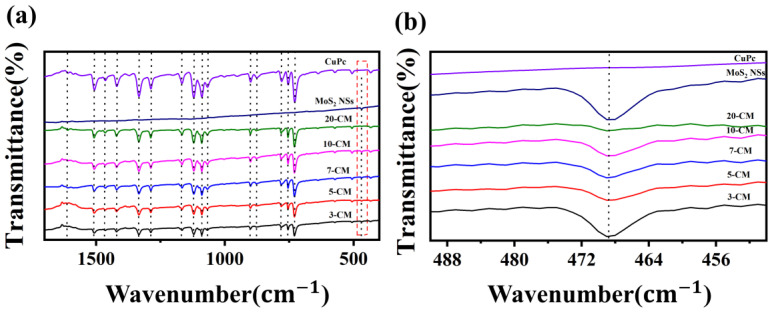
(**a**) Fourier transform infrared spectra of CuPc, MoS_2_ NSs, 3-CM, 5-CM, 7-CM, 10-CM, and 20-CM. (**b**) Local magnification of figure (**a**).

Figure 6 shows the FTIR spectra of CuPc, MoS_2_ NSs, 3-CM, 5-CM, 7-CM, 10-CM, and 20-CM. In Figure 6a, the spectra of MoS_2_ nanosheets, 3-CM, 5-CM, 7-CM, 10-CM, and 20-CM all showed an absorption peak at 468 cm^−1^. The magnification in Figure 6b shows that the intensity of the MoS_2_ absorption peak increased with the MoS_2_-to-CuPc ratio. Figure 6a also shows that CuPc, 3-CM, 5-CM, 7-CM, 10-CM, and 20-CM all contained similar absorption peaks, whose intensity decreased upon increasing the MoS_2_-to-CuPc ratio. Among them, the peaks at 726 cm^−1^, 752 cm^−1^, and 780 cm^−1^ belonged to the -C-H- out-of-plane bending vibration, and the peaks at 874 cm^−1^, 900 cm^−1^, 1065 cm^−1^, 1086 cm^−1^, and 1119 cm^−1^ belonged to -C-C-telescopic vibrations. The peaks at 1166 cm^−1^ and 1333 cm^−1^ belonged to -C-O- telescopic vibration. The peak at 1285 cm^−1^ belonged to -O-H-in-plane telescopic vibrations. The peaks at 1418 cm^−1^, 1465 cm^−1^, 1507 cm^−1^, and 1611 cm^−1^ belonged to telescopic vibrations of the -C=C- bonds in the phthalocyanine skeleton [30].

### 3.2. Gas-Sensing Studies

**Figure 7:**
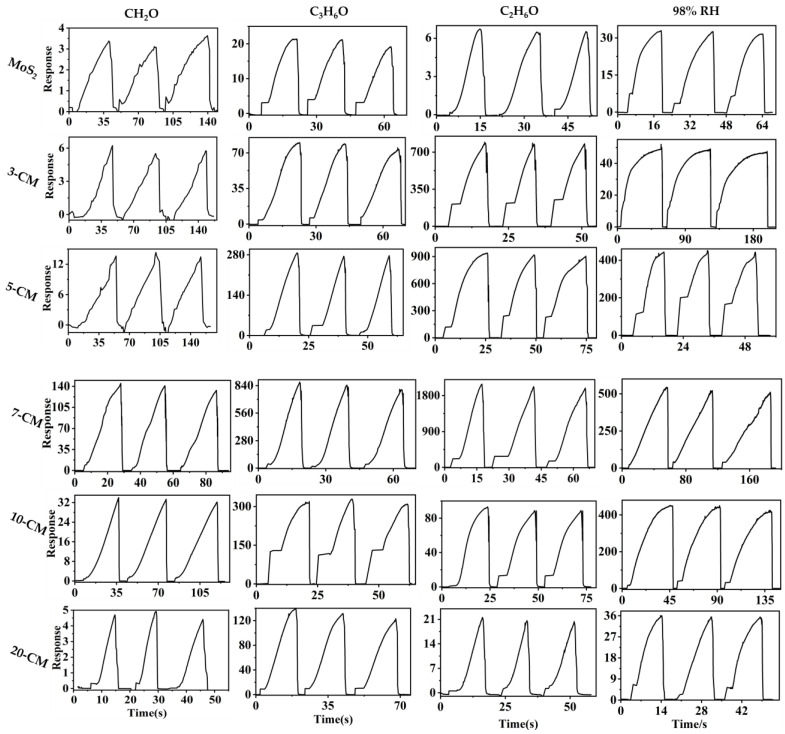
Response curves of MoS_2_-, 3-CM-, 5-CM-, 7-CM-, 10-CM-, and 20-CM-based sensors to 1000 ppm CH_2_O, C_3_H_6_O, C_2_H_6_O, and 98% RH at room temperature.

**Figure 8:**
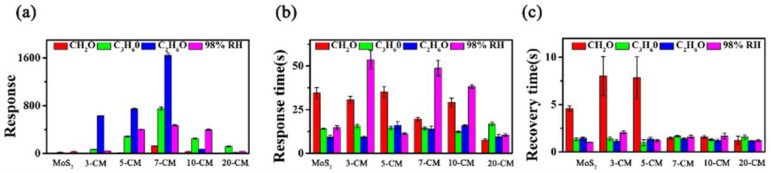
(**a**) Mean response; (**b**) response time; and (**c**) recovery time for MoS_2_, 3-CM, 5-CM, 7-CM, 10-CM, and 20-CM sensors to 1000 ppm CH_2_O, C_3_H_6_O, C_2_H_6_O, and 98% RH.

The gas-sensing performance of gas sensors based on the 2D-MoS_2_/1D-CuPc heterojunction was studied using a homemade gas-sensing detection platform. Figure 7 shows the effect of the MoS_2_-to-CuPc ratio on the gas-sensing response. The response curve shows that the 2D-MoS_2_/1D-CuPc heterojunction with different doping ratios had a relatively stable response to 1000 ppm of formaldehyde (CH_2_O), acetone (C_3_H_6_O), ethanol (C_2_H_6_O), and 98% RH at room temperature. The response of the 2D-MoS_2_/1D-CuPc heterojunction to the four target gases first increased, and then, decreased as the proportion of CuPc increased. Figure 8a shows that the best gas-sensing performance was obtained when the ratio of MoS_2_ to CuPc was 20:7. Two-dimensional MoS_2_/one-dimensional CuPc had the highest sensitivity toward C_2_H_6_O. The average responses of pure MoS_2_ to 1000 ppm CH_2_O, C_3_H_6_O, C_2_H_6_O, and 98% RH were 2.9, 18.4, 5.3, and 31.1, respectively. For 3-CM, the mean response of 1000 ppm CH_2_O, C_3_H_6_O, C_2_H_6_O, and 98% RH increased to 5.2, 69.5, 630.6, and 43.3, respectively. The mean responses of 5-CM toward 1000 ppm CH_2_O, C_3_H_6_O, C_2_H_6_O, and 98% RH were 12.4, 285.1, 745.5, and 400.7, respectively. The average responses of 7-CM to 1000 ppm CH_2_O, C_3_H_6_O, C_2_H_6_O, and 98% RH were 125.6, 753.1, 1645.1, and 471.7, respectively. Compared with pure MoS_2_, the response of 7-CM to 1000 ppm CH_2_O, C_3_H_6_O, C_2_H_6_O, and 98% RH increased by 122.7, 734.6, 1639.8, and 440.5, respectively. When the ratio of MoS_2_ to CuPc further decreased, the sensitivity of 2D-MoS_2_/1D-CuPc began to decrease. The results showed that the responses of 10-CM to 1000 ppm CH_2_O, C_3_H_6_O, C_2_H_6_O, and 98% RH were 30.9, 251.4, 72.9, and 396.8, respectively, which were all better than those of pure MoS_2_. The responses of 20-CM to 1000 ppm CH_2_O, C_3_H_6_O, C_2_H_6_O, and 98%RH were 4.2, 117.9, 16.8, and 32.1, respectively, which were also better than those of pure MoS_2_. These results show that the ratio of MoS_2_ and CuPc had a significant effect on the gas sensitivity of the 2D-MoS_2_/1D-CuPc heterojunction.

When the 2D-MoS_2_/1D-CuPc heterojunction sensor was exposed to C_2_H_6_O, C_3_H_6_O, and CH_2_O gas, surface-adsorbed oxygen ($O_{2}^{-}$ < 100 °C, $O_{2}^{-}$ 100–300 °C, $O_{2}^{-}$ > 300 °C) reacted with C_2_H_6_O, C_3_H_6_O, and CH_2_O as follows:

(1) $$C_{2}H_{6}O\left(g\right)+3O_{2}^{-}\left(s\right)\to 2CO_{2}+3H_{2}O+3e^{-}$$

(2) $$C_{3}H_{6}O\left(g\right)+4O_{2}^{-}\left(s\right)\to 3CO_{2}+3H_{2}O+4e^{-}$$

(3) $$CH_{2}O\left(g\right)+O_{2}^{-}\left(s\right)\to CO_{2}+H_{2}O+e^{-}$$

In the above reaction [32,33,34], e^−^ represents electron conduction, and $O_{2}^{-}\left(s\right)$ represents surface-adsorbed oxygen ions. $C_{2}H_{6}O\left(g\right)$, ${\;C}_{3}H_{6}O\left(g\right)$, and$\;CH_{2}O\left(g\right)$, respectively represent adsorbed C_2_H_6_O, C_3_H_6_O, and CH_2_O molecules. The reduced gas molecules released electrons into 2D-MoS_2_/1D-CuPc during the reaction, and the current of 7-CM increased rapidly, indicating a good synergistic effect between the materials at this ratio.

To further evaluate the effect of the ratio of MoS_2_ and CuPc on the sensing performance of 2D-MoS_2_/1D-CuPc, the average response size, response time, and recovery time of the six sensors to four target analytes was analyzed. Figure 8b,c show that the response of the 2D-MoS_2_/1D-CuPc heterojunction to the analyte increased first, and then, decreased as the mass ratio of MoS_2_ to CuPc decreased. The response time of all sensors was less than 60 s, and the recovery time was less than 10 s. This fast recovery was due to the good crystallinity of the sensitive structural elements, which provided the gas sensors with greater electron mobility. The one-dimensional structure facilitated the transport and transfer of electrons, as well as rapid response and recovery.

**Figure 9:**
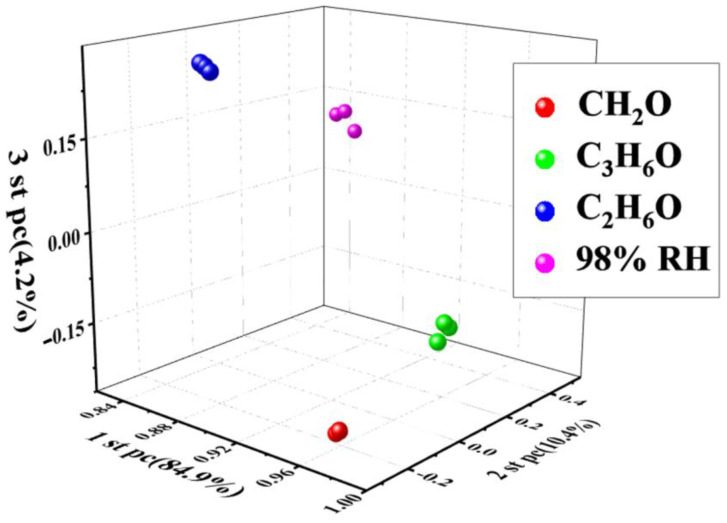
Three-dimensional PCA plot derived from the average response of MoS_2_, 3-CM, 5-CM, 7-CM, 10-CM, and 20-CM sensors.

A significant advantage of this sensor array over a single-sensor array is that an unknown analyte can be identified and detected based on mathematical analyses, kinetics, and thermodynamics [35] or via principal component analysis (PCA) [36,37]. The sensor array was made of 2D-MoS_2_/1D-CuPc with different MoS_2_/CuPc ratios. The recognition performance of the sensor array was further evaluated using 3D PCA (Figure 9). The coordinates of the four test samples could be distinguished, and the sensor identified all four samples. The results show that the 2D-MoS_2_/1D-CuPc heterojunction had high sensitivity and selectivity.

**Figure 10:**
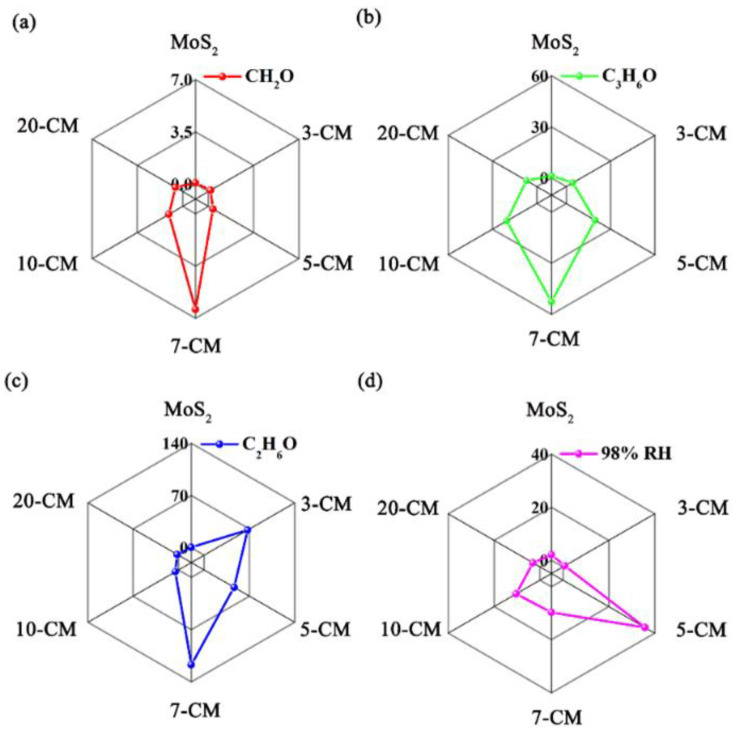
Characteristic fingerprints derived from kinetic and thermodynamic parameters from (**a**) CH_2_O, (**b**) C_3_H_6_O, (**c**) C_2_H_6_O, and (**d**) 98% RH at 1000 ppm at room temperature.

To further investigate the gas-sensing properties of the sensor array, its thermodynamic and kinetic parameters were combined, converted into a graphical signal, and combined with image recognition technology. The reaction amplitude and reaction time were taken as the thermodynamic and kinetic parameters, respectively. Six sensors showed six reaction amplitudes and six reaction times for each analyte. The ratio of the six reaction values to the six reaction times yielded six new thermodynamic and kinetic parameters, which were used to construct visually distinct hexagons, as shown in Figure 10. This shows that the sensor array had high recognition performance.

**Figure 11:**
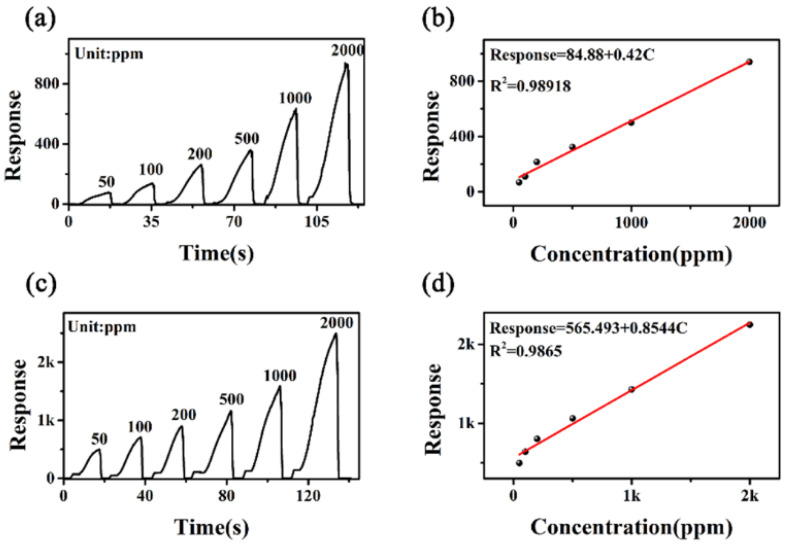
(**a**) Sensing curves of the 7-CM sensor to different concentrations of C_3_H_6_O; (**b**) fitted plots of the response vs. C_3_H_6_O concentration; (**c**) sensing curves of the 7-CM sensor to different concentrations of C_2_H_6_O; (**d**) fitted plots of the response vs. C_2_H_6_O concentration.

To study the real-time monitoring performance of 7-CM, dynamic tests with different concentrations of C_3_H_6_O and C_2_H_6_O were carried out. The relationship between response and vapor concentration at room temperature is shown in Figure 11. Figure 11a,c show that the surface C_3_H_6_O and C_2_H_6_O vapors of 7-CM had good gas-sensing characteristics. Figure 11b,d demonstrate that 7-CM had good linearity toward C_3_H_6_O and C_2_H_6_O vapors. The device had outstanding advantages in terms of quantitative detection, direct readout, simplified correction, and auxiliary circuitry.

To evaluate the performance of 2D-MoS_2_/1D-CuPc heterojunction-based sensors, Table 1 compares the sensing performance of C_2_H_6_O sensors with different sensing materials. Three-dimensional (MoS_2_)/ZnO and MoS_2_/TiO_2_ detected C_2_H_6_O, but their response time and recovery time were longer than those of 7-CM and MoS_2_/CeO_2_. MoS_2_/NiCo_2_O_4_ and CdS/MoS_2_ exhibited a faster response and recovery, but they only operated at temperatures above 100 °C, which means higher power consumption and a more complex operating environment. Compared with these composites, the 2D-MoS_2_/1D-CuPc heterojunction showed higher responses and shorter response times at room temperature. Especially, the responses of the 2D-MoS_2_/1D-CuPc composite were better than all the sensing materials in Table 1, showing good application potential.

## 4. Analysis of Gas-Sensing Mechanism

**Figure 12:**
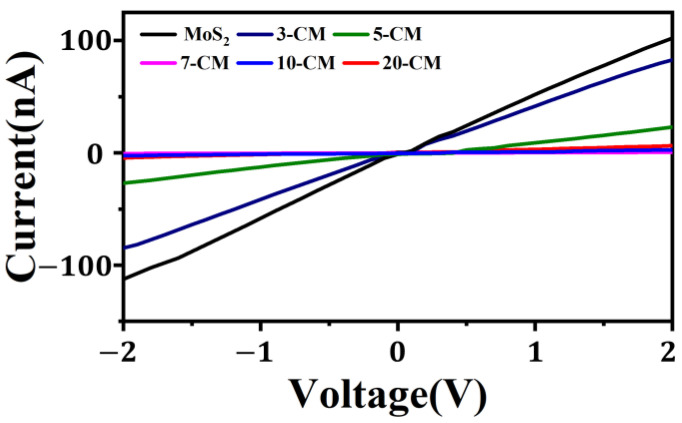
*I–V* curves of MoS_2_-, 3-CM-, 5-CM-, 7-CM-, 10-CM-, and 20-CM-based sensors.

Figure 12 shows the current–voltage (*I-V*) characteristic curves of MoS_2_, 3-CM, 5-CM, 7-CM, 10-CM, and 20-CM sensors, in which the conductivity of 3-CM to 7-CM decreased sequentially. This was attributed to an increase in the heterojunction barrier between MoS_2_ nanosheets and CuPc micro-nanowires. In addition, as the mass ratio of CuPc increased, the electrical conductivity of the material decreased due to the lower electrical conductivity of CuPc compared with MoS_2_. The 7-CM surface contained dispersed CuPc micro-nanowires that ensured the penetration and diffusion of gas molecules, which improved the sensitivity of the sensor [46].

**Figure 13:**
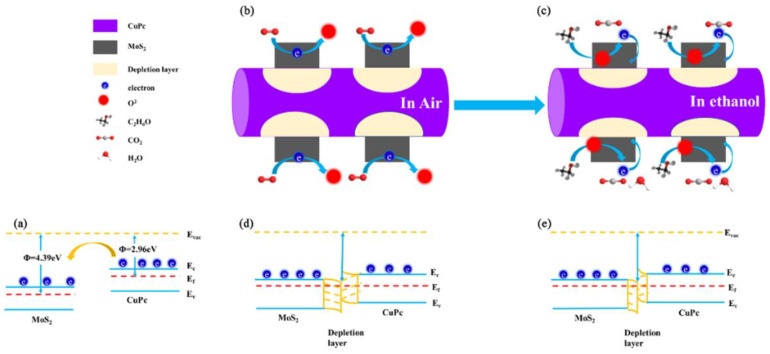
Schematic diagrams of (**a**) the band structure of MoS_2_ and CuPc; (**b**) the gas-sensing mechanism of MoS_2_/CuPc composites in the air; (**c**) the gas-sensing mechanism of MoS_2_/CuPc composites in ethanol; (**d**) the band structure of MoS_2_/CuPc composites in the air; (**e**) the band structure of MoS_2_/CuPc composites in ethanol.

Enhancements in the gas-sensing performance of the 2D-MoS_2_/1D-CuPc heterojunction were attributed to the heterojunction between MoS_2_ and CuPc. CuPc is a p-type semiconductor, while MoS_2_ is an n-type semiconductor. Figure 13a shows a schematic diagram of the band structure of MoS_2_ and CuPc, where E_f_ and Φ are the Fermi level and work function, respectively. The work functions of MoS_2_ and CuPc were 4.39 eV and 2.96 eV, respectively. Figure 13a shows that CuPc had a higher Fermi level than MoS_2_, allowing electrons to quickly transfer from CuPc to MoS_2_. Thus, the bands of CuPc and MoS_2_ began to shift until their Fermi levels reached a new equilibrium (Figure 13d) and generated a built-in Schottky barrier (*qV_0_*). Thus, a depletion layer existed at the interface between MoS_2_ and CuPc [47]. When the 2D-MoS_2_/1D-CuPc composite contacted the target gas (e.g., ethanol), electron exchange occurred (Figure 13c). As a result, the conductivity of the MoS_2_/CuPc heterojunction was greatly increased, which improved its response. In addition, the CuP_C_ micro-nanowires on the surface of the MoS_2_ nanosheets acted as a p-type dopant and increased the specific surface area of the 2D-MoS_2_/1D-CuPc heterojunction, which provided more adsorption sites for target gas molecules, thus improving its sensitivity. The enhanced charge transfer and increased number of active sites improved the sensing performance of the 2D-MoS_2_/1D-CuPc heterojunction.

## 5. Conclusions

We prepared 2D-MoS_2_/1D-CuPc heterojunction composites with different mass ratios of MoS_2_ to CuPc via a heating/magnetic reflux stirring method, and then, used them to construct 2D-MoS_2_/1D-CuPc-based gas sensors. The results showed that the response of the 2D-MoS_2_/1D-CuPc heterojunction to gas analytes was related to the mass ratio of MoS_2_ to CuPc. When the ratio of MoS_2_ to CuPc was 20:7, the best gas-sensing performance of the 2D-MoS_2_/1D-CuPc heterojunction was obtained. Compared with the MoS_2_ sensor, the responses of 7-CM to 1000 ppm CH_2_O, C_3_H_6_O, C_2_H_6_O, and 98% RH were increased by 122.7, 734.6, 1639.8, and 440.5, respectively, at room temperature. According to PCA and radar analysis, a 2D-MoS_2_/1D-CuPc heterojunction sensor array could identify and detect four target gases. After further data processing, the sensor array showed higher recognition ability. These results provide an experimental reference for the development of high-performance MoS_2_-based gas sensors.

## Figures and Tables

**Table 1:** Comparison of C_2_H_6_O-sensing performance using different sensors.

Materials	Working Temperature	Concentration (ppm)	Response	Res/Rec Time (s)	Ref.
SnO_2_QD/MoS_2_	180 °C	50	0.49	25/85	[38]
MoS_2_/CeO_2_	RT	50	7.78	7/5	[39]
3D(MoS_2_)/ZnO	220 °C	500	12.08	30/10	[40]
MoS_2_/TiO_2_	350 °C	100	0.62	52/155	[41]
MoS_2_/PSi	RT	40	0.17	---	[42]
MoS_2_/NiCo_2_O_4_	170 °C	100	9.00	15/6	[43]
MoS_2_Nanosheets	RT	114	1.14	2.4/29	[44]
CdS/MoS_2_	210 °C	100	0.95	20/9	[45]
2D-MoS_2_/1D-CuPc	RT	100	710.42	<10/<4	This work
		50	497.69		

## Data Availability

The data presented in this study are available on request from the corresponding author.

## Supplementary Materials

The following supporting information can be downloaded at: https://www.mdpi.com/article/10.3390/s23239321/s1, Figure S1: (a) Schematic of synthesis of MoS_2_ nanosheets; (b) Schematic of the preparation process of CuPc micro-nanowires; (c) Schematic of the preparation process of the 2D-MoS2/1D-CuPc based gas sensor; Figure S2: (a) XPS survey spectra of MoS_2_, CuPc, and 7-CM; (b) Cu 2p_1/2_, Cu 2p_3/2_ spectra of CuPc and 7-CM; (c) C 1s spectra of MoS_2_; (d) C 1s spectra of CuPc; (e) C 1s spectra of 7-CM; (f) O 1s spectra of MoS_2_; (g) O 1s spectra of CuPc; (h) O 1s spectra of 7-CM; (i) MoS_2_ S2p_1/2_, S2p_3/2_, and S-C spectra; (j) 7-CM S2p_1/2_ and S2p_3/2_ spectra; (k) Mo^4+^ 3d_3/2_, Mo4+ 3d_5/2_ spectra of MoS_2_; (l) Mo^4+^ 3d_3/2_, Mo^4+^ 3d_5/2_, and S 2s spectra of 7-CM; (m) N 1s spectrum of CuPc; (n) C-NH_2_, C-N, N 1s, N-Cu (N ions in CuPc), Mo 3p_3/2_ spectra of 7-CM.
