# Supplementary material for: Preparation and Gas-Sensing Properties of Two-Dimensional Molybdenum Disulfide/One-Dimensional Copper Phthalocyanine Heterojunction
Source: Sensors (Basel). 2023 Nov 22;23(23):9321. doi: 10.3390/s23239321 (PMC10708874; doi:10.3390/s23239321)
Supplement: Supplementary file 1 [file sensors-23-09321-s001.zip › sensors-2707469-supplementary.pdf]

Preparation and gas-sensing properties of 2D-MoS_2/1D-CuPc heterojunction

Guoqing Chen [1], Xiaojie Xu [1], Hao Wang [1] and Talgar Shaymurat [1,*]

[1] Key Laboratory of New Energy and Materials Research, Xinjiang Institute of Engineering, Urumqi, Xinjiang 830023, China; cgq08250624@163.com (G.C.);18099415421@163.com (X.X.);wh18299190475@163.com (H.W.)

* Correspondence: talgar.shaymurat@vip.163.com

(Guoqing Chen and Xiaojie Xu are co-first authors of the article)

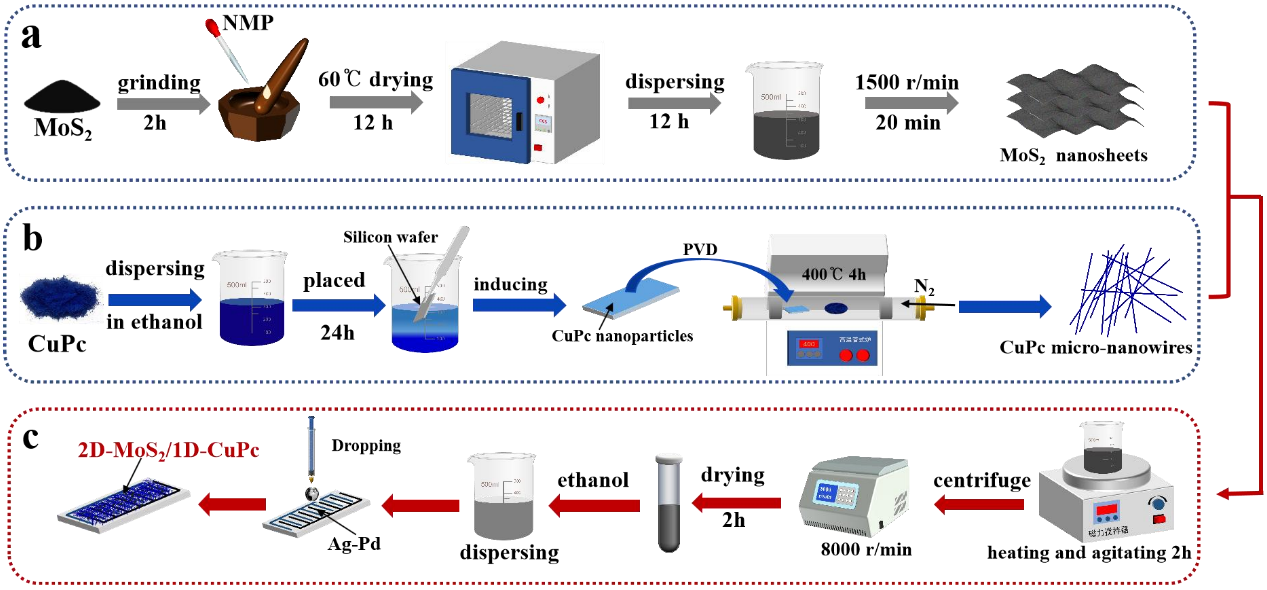

Figure S1. (a) Schematic of synthesis of MoS_2 nanosheets; (b) Schematic of the preparation process of CuPc micro-nanowires; (c) Schematic of the preparation process of the 2D-MoS_2/1D-CuPc based gas sensor

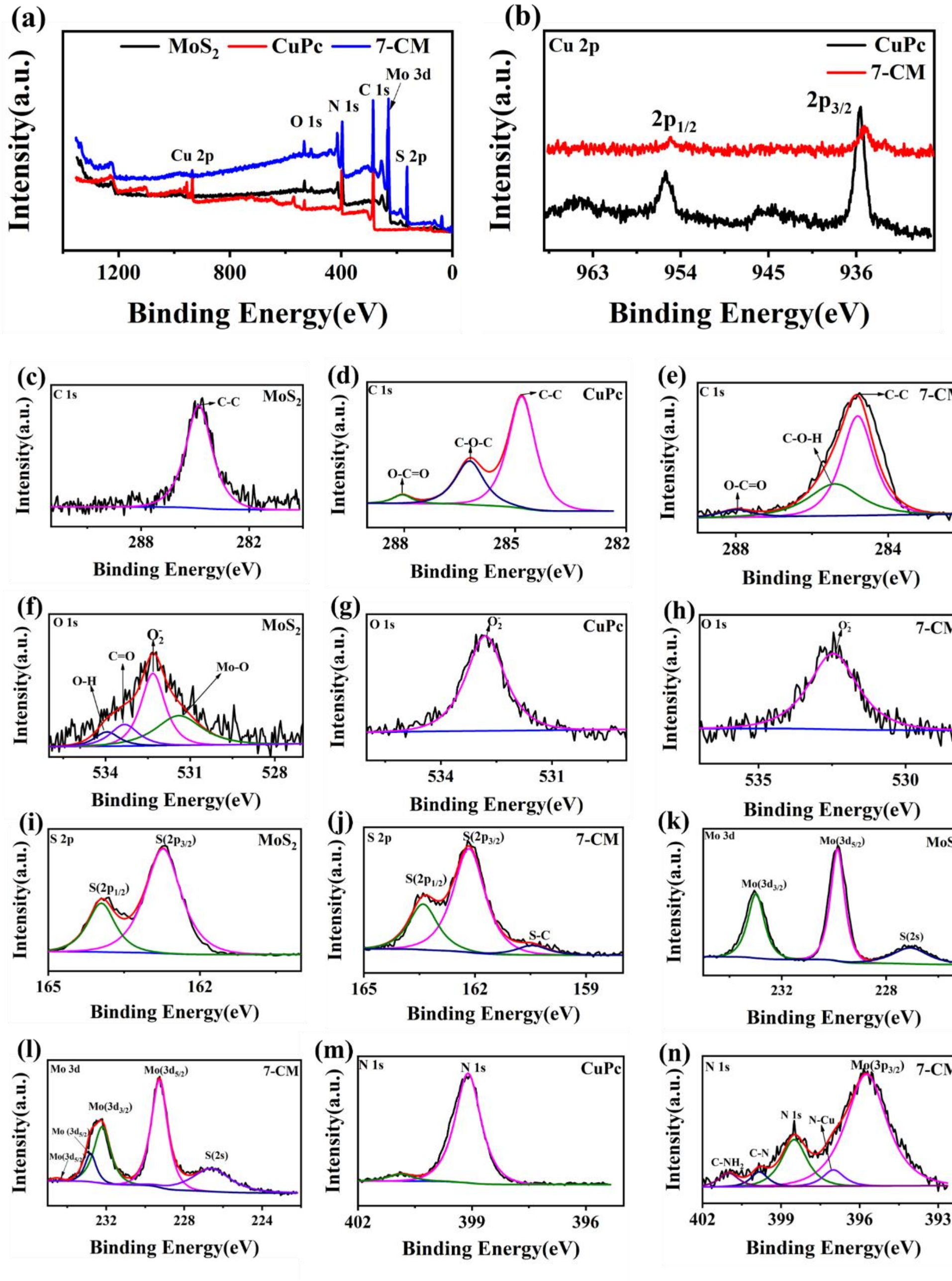

Figure S2. (a) XPS survey spectra of MoS_2, CuPc, and 7-CM; (b) Cu $2p_{1/2}$, Cu $2p_{3/2}$ spectra of CuPc and 7-CM; (c) C 1s spectra of MoS_2; (d) C 1s spectra of CuPc; (e) C 1s spectra of 7-CM; (f) O 1s spectra of MoS_2; (g) O 1s spectra of CuPc; (h) O 1s spectra of 7-CM; (i) MoS_2 $S2p_{1/2}$, $S2p_{3/2}$, and S-C spectra; (j) 7-CM $S2p_{1/2}$ and $S2p_{3/2}$ spectra; (k) Mo^{4+} $3d_{3/2}$, Mo^{4+} $3d_{5/2}$ spectra of MoS_2; (l) Mo^{4+} $3d_{3/2}$, Mo^{4+} $3d_{5/2}$, and S 2s spectra of 7-CM; (m) N 1s spectrum of CuPc; (n) C-NH_2, C-N, N 1s, N-Cu (N ions in CuPc), Mo $3p_{3/2}$ spectra of 7-CM
